# Natural products and phytochemical nanoformulations targeting mitochondria in oncotherapy: an updated review on resveratrol

**DOI:** 10.1042/BSR20200257

**Published:** 2020-04-03

**Authors:** Milad Ashrafizadeh, Sara Javanmardi, Masoumeh Moradi-Ozarlou, Reza Mohammadinejad, Tahereh Farkhondeh, Saeed Samarghandian, Manoj Garg

**Affiliations:** 1 Department of Basic Science, Faculty of Veterinary Medicine, University of Tabriz, Tabriz, Iran; 2 Department of Clinical Sciences, Faculty of Veterinary Medicine, University of Tabriz, Tabriz, Iran; 3 Department of Pathobiology, Faculty of Veterinary Medicine, University of Tabriz, Tabriz, Iran; 4 Neuroscience Research Center, Institute of Neuropharmacology, Kerman University of Medical Sciences, Kerman, Iran; 5 Cardiovascular Diseases Research Center, Birjand University of Medical Sciences, Birjand, Iran; 6 Healthy Ageing Research Center, Neyshabur University of Medical Sciences, Neyshabur, Iran; 7 Department of Basic Medical Sciences, Neyshabur University of Medical Sciences, Neyshabur, Iran; 8 Amity Institute of Molecular Medicine and Stem cell Research (AIMMSCR), Amity University, Noida, Uttar Pradesh 201313, India

**Keywords:** Cancer therapy, NF-kB signaling, reactive oxygen species, Resveratrol, Signaling pathway

## Abstract

Mitochondria are intracellular organelles with two distinct membranes, known as an outer mitochondrial membrane and inner cell membrane. Originally, mitochondria have been derived from bacteria. The main function of mitochondria is the production of ATP. However, this important organelle indirectly protects cells by consuming oxygen in the route of energy generation. It has been found that mitochondria are actively involved in the induction of the intrinsic pathways of apoptosis. So, there have been efforts to sustain mitochondrial homeostasis and inhibit its dysfunction. Notably, due to the potential role of mitochondria in the stimulation of apoptosis, this organelle is a promising target in cancer therapy. Resveratrol is a non-flavonoid polyphenol that exhibits significant pharmacological effects such as antioxidant, anti-diabetic, anti-inflammatory and anti-tumor. The anti-tumor activity of resveratrol may be a consequence of its effect on mitochondria. Multiple studies have investigated the relationship between resveratrol and mitochondria, and it has been demonstrated that resveratrol is able to significantly enhance the concentration of reactive oxygen species, leading to the mitochondrial dysfunction and consequently, apoptosis induction. A number of signaling pathways such as sirtuin and NF-κB may contribute to the mitochondrial-mediated apoptosis by resveratrol. Besides, resveratrol shifts cellular metabolism from glycolysis into mitochondrial respiration to induce cellular death in cancer cells. In the present review, we discuss the possible interactions between resveratrol and mitochondria, and its potential application in cancer therapy.

## Introduction

Naturally occurring compounds are beneficial in the treatment of pathological events due to their excellent therapeutic and biological activities [1,2]. It is suggested that plant-derived chemicals are less harmful with minimal toxicity [3,4]. Resveratrol is a naturally occurring polyphenol exclusively found in a number of fruits and vegetables such as blueberries, grape, and peanut [5]. This important compound has a variety of pharmacological and health-promoting impacts such as antioxidant [6], anti-inflammatory [7], anti-tumor [8], anti-diabetic [9], hepatoprotective [10], neuroprotective [11] and cardioprotective [12], and is capable of improving dyslipidemia [13]. These therapeutic impacts of resveratrol have led to its extensive application in the treatment of various disorders. Accumulating data demonstrates that resveratrol is able to target multiple molecular signaling pathways to exert its therapeutic impacts. For instance, in term of anti-tumor activity, resveratrol is capable of inhibiting the molecular pathways involved in the invasion and malignancy of cancer cells. Administration of resveratrol is associated with the down-regulation of the CXCR4 signaling pathway, leading to the inhibition of epithelial-to-mesenchymal transition (EMT) [14]. Resveratrol is advantageous in improving the potential of chemotherapy. Paclitaxel (PTX) is one of the most common chemotherapeutic agents in cancer therapy. However, the resistance of cancer cells is related to its reduced anti-tumor activity. Treatment of cancer cells with resveratrol sensitizes cancer cells to PTX therapy [15]. Besides, resveratrol up-regulates the expression of DUSP1 to enhance the efficacy of cisplatin in chemotherapy and eradication of prostate cancer cells [16]. Resveratrol can be considered as a promising candidate in the treatment of neurological disorders (NDs). A newly published article has questioned the role of resveratrol in the alleviation of cognitive deficits. Aggregations of amyloid-beta (Aβ) plaques and microtubule-associated protein tau significantly enhance the chance of Alzheimer’s disease (AD) development. There have been attempts to reduce or inhibit this aggregation. By suppressing the accumulation of protein tau, Res remarkably diminishes the cytotoxicity and inhibits neuroinflammation, leading to the amelioration of memory deficits [17–19].

Diabetes mellitus (DM) is a chronic metabolic disorder associated with an increase in the levels of serum glucose [20]. This disorder negatively affects various parts of the body demanding immediate management and treatment [21]. Resveratrol has demonstrated great potential in the treatment of DM. Insulin resistance is one of the most challenging difficulties related to DM. Down-regulation of SNARE protein remarkably enhances insulin resistance [22]. Resveratrol-loaded solid lipid nanoparticles (Res-SLNs) are able to reduce insulin resistance. Administration of Res-SLNs significantly decreased oxidative stress and inhibited weight loss. Investigation of molecular signaling pathways demonstrated that Res-SLNs are capable of the up-regulation of SNARE resulting in attenuation of DM [23]. DM harmfully affects reproductive function. Based on the therapeutic effects of resveratrol, its administration can be advantageous in DM treatment. Resveratrol remarkably increases the motility and viability of sperm and promotes DNA integrity during DM [24]. These studies support the protective effects of resveratrol against human diseases.

## Resveratrol

In spite of having great pharmacological effects, there are a number of factors that limit the therapeutic impacts of resveratrol. This compound has poor bioavailability after oral administration [25,26], restricting its activities. Rapid metabolism and low solubility are two factors responsible for the poor bioavailability of resveratrol [27,28]. For *in vivo* experiments, resveratrol is dissolved in 10% ethanol [29,30] and for *in vitro* purposes, resveratrol is dissolved in 0.2% DMSO [31,32]. Due to the unexpected harmful effects of resveratrol in 0.2%, DMSO on cells and tissues, resveratrol and DMSO combination is of interest in experiments related to the cultured cells (*in vitro*) [33,34]. So, it is obvious that poor bioavailability of resveratrol not only has limited its therapeutic and biological impacts, but also has led to some effects on the administration route of resveratrol. A variety of attempts have been performed to improve the bioavailability of resveratrol and among them, the nano-based drug delivery systems are of importance [35]. In a newly published article, Thipe and colleagues synthesized resveratrol-loaded gold nanoparticles to enhance the anti-tumor potential of resveratrol against breast, pancreatic and prostate cancers. The findings demonstrated that the enhanced corona of resveratrol on gold nanoparticles significantly promotes the bioavailability of resveratrol, leading to the reduced viability and proliferation of cancer cells [36]. Besides, resveratrol-loaded lipid-core nanocapsules have higher antioxidant and anti-inflammatory activities compared to the resveratrol alone [37]. Overall, polymeric nanoparticles [38,39], solid lipid nanoparticles [40,41], self-emulsifying drug delivery systems [42,43], nanoemulsions [44,45], liposomes [46,47], nanosuspensions [48] and nanofibers [49] have been developed for delivery of resveratrol and the results have been satisfactory.

## Resveratrol in the treatment of cancer

Resveratrol has demonstrated great potential in the treatment of various cancers due to its capability in targeting various molecular pathways [50–54]. By inhibition of TNF-receptor associated factor 6 (TRAF6), resveratrol inhibits EMT to reduce the invasion of prostate cancer cells [55]. In colon cancer cells, resveratrol-loaded solid lipid nanoparticles remarkably stimulate apoptotic cell death to diminish the growth and proliferation of cancer cells [56]. In addition to apoptosis, resveratrol induces cell cycle arrest in cancer cells [57,58]. Due to its demethylation capability, resveratrol enhances the sensitivity of anaplastic thyroid cancer cells to retinoic acid [59]. Notably, resveratrol can be applied in cancer immunotherapy. Programmed death-1 (PD-1) is associated with reduced severity of the immune system during cancer progression. By administration of resveratrol, PD-1 is accumulated in endoplasmic reticulum and its stabilization is inhibited, leading to the increased anti-tumor activity of T-cells [60].

Resveratrol administration is advantageous in the treatment of colon cancer by triggering signaling pathways that can lead to the stimulation of apoptosis. A combination of resveratrol and As_2_O_3_ diminishes the expression of hEGR channels on the cell membrane. This results in the dissociation of hERG and integrin β1 to induce apoptosis in colon cancer cells [61]. Furthermore, resveratrol has shown great potential in the treatment of oral cancer. Thyroxine is associated with enhanced proliferation and progression of oral cancer cells by down-regulation of apoptotic factor BAD and up-regulation of PD-1. Resveratrol exerts an inhibitory effect on the function of thyroxine so that resveratrol supplementation enhances the expression of BAD and inhibits PD-1 to suppress oral cancer cells [62]. By down-regulation of the snail signaling pathway, resveratrol reduces EMT to inhibit the invasion of colon cancer cells [63]. It is worth mentioning that resveratrol activates tumor suppressor Rad9 to induce premature senescence in lung and breast cancer cells [64]. Another published article also demonstrates the capability of resveratrol in the regulation of epigenetic factors [65]. These studies reveal the potential activity of resveratrol as an anti-tumor agent.

Overall, triggering reactive oxygen species (ROS) production is a promising strategy in cancer therapy, since these reactive species are involved in stimulation of apoptosis through mitochondrial dysfucntion and much attention has been directed toward enhancing the ROS generation in cancer cells to suppress their malignancy and proliferation [66,67]. To date, there has been no article to investigate the relationship between the inhibitory effect of resveratrol on ROS production and its anti-tumor activity. However, the accumulating data have shown that the major mechanism for the antioxidant activity of resveratrol is related to its effect on the nuclear factor erythroid 2-related factor 2 (Nrf2) signaling pathway. Resveratrol, by activation of the Nrf2 signaling pathway, ameliorates oxidative stress [68] to improve colitis [69], arthritis [63], diabetes [70] and non-alcoholic fatty liver disease [71].

## Mitochondria at a glance

Mitochondria are one of the most important intracellular organelles with the size as low as one micron [72,73]. They are available in two characteristic forms including granular form and filamentary form. Originally, mitochondria have been derived from aenobic α-proteobacteria. As a consequence, it has been demonstrated that a variety of mitochondrial proteins have bacterial origin [74]. The number of mitochondria varies in each cell and exists in all eukaryotic cells except mature erythrocytes. Due to the bacterial origin of mitochondria, this important organelle has two distinct membranes known as the outer mitochondrial membrane (OMM) and inner mitochondrial membrane (IMM). Notably, each of these membranes has its own functions. The IMM shrivels inwards resulting in the formation of folds known as cristae that surround the matrix. However, OMM has a more vital function. OMM is responsible for the exchange of metabolites by its pores through passive diffusion and translocases located on the OMM. Besides, OMM protects the cytosol against damages such as degradation by inhibition of releasing ROS and mitochondrial DNA (mtDNA). Mitochondria are well-known as the powerhouse of the cell and contribute to the production of adenosine triphosphate (ATP). The mitochondria generate energy in the form of ATP via oxidative phosphorylation pathway. Regardless of the essential function of mitochondria in providing energy, this intracellular organelle significantly diminishes the levels of oxygen in the cell by consuming it in energy production, leading to a substantial decrease in the concentration of this potentially toxic substance.

## Mitochondria and apoptosis

Although much emphasis has been directed towards the role of mitochondria in providing energy, mitochondria play a significant role in maintaining homeostasis [75,76]. The structure of mitochondria is of importance in induction or inhibition of apoptotic cell death [77,78]. Accumulating data demonstrates that mitochondria are the central gateway of the intrinsic pathway of apoptosis [79,80]. This reveals the potential role of mitochondria in the regulation of life and death [81,82]. The beginning point in the stimulation of apoptotic cell death by mitochondria is the release of cytochrome *C* (Cyt C) into the cytosol. This occurs after the mitochondrial outer membrane permeabilization (MOMP) [83,84]. The lethal functions are controlled by MOMP during apoptotic cell death that is mediated via the proteins involved in the formation of pores including pro-apoptotic proteins include B-cell lymphoma 2 (Bcl-2), Bcl-2 associated x protein (Bax) and Bcl-2 antagonist/killer protein (Bak) [85]. Increasing evidence shows that mitochondria contribute to necrotic cell death and are able to disrupt Ca^2+^ homeostasis and ROS production [86,87]. In addition to the Cyt C, other molecules such as Samc/Diablo and endonuclease G are released into the cytosol. The release of cyt C into cytosol occurs in response to the stress conditions such as high ROS concentration and DNA damage [88]. Next, cyt C leads to the formation of apoptosome containing procaspase-9 and apoptotic protease activating factor 1 (Apaf-1). Consequently, caspase-9 stimulates caspase cascade, resulting in apoptosis. Caspase-3 is one of the targets of caspase-9. After the generation of caspase-3 from pro-caspase-3 by the stimulation of caspase-9, the caspase-3 induces morphological alterations of apoptosis in cells such as chromatin condensation, DNA fragmentation and blebbing to direct the cell toward apoptotic cell death [89]. Noteworthy, caspase-9 also activates caspase-7 and an increase occurs in both caspase-7 and -3. Caspase-7 along with caspase-3 mediates the induction of apoptosis in target cells [90]. The cells undergoing apoptosis have a number of morphological signs such as membrane blebbing, cell shrinking, chromatin condensation and apoptotic bodies [91,92].

## Mitochondria and cancer

Obviously, mitochondrial dysfunction is associated with a variety of pathological conditions such as NDs [93], hepatic disorders [69] and so on. This is due to the potential role of mitochondria in the induction of apoptotic cell death. However, this intracellular organelle can be targeted as a therapeutic candidate in cancer therapy. Although much attention has been made in the regulation of mitochondrial-mediated apoptosis and preserving mitochondrial homeostasis to prevent the development of pathological conditions, it seems that the administration of agents targeting mitochondria is of importance in cancer therapy. Orientin is a naturally occurring flavonoid with great pharmacological impacts, particularly anti-tumor activity [94,95]. It is held that the administration of orientin is of interest in the treatment of colorectal cancer. Orientin induces apoptotic cell death in colorectal cancer cells. By activation of pro-apoptotic proteins, orientin disrupts the integrity of OMM to release cyt C and Smac/Diablo into cytosol resulting in stimulation of mitochondrial-mediated apoptosis [96]. Accumulating data demonstrates that breast cancer is one of the most common malignancies in women worldwide [97]. It has been demonstrated that the conventional therapeutic strategies such as chemotherapy and radiotherapy are no longer efficient due to the resistance of breast cancer cells [98,99]. Administration of plant-derived chemicals can sensitize cancer cells into radiotherapy and chemotherapy. Berberine is a potent anti-tumor agent and 13-ethylberberine (13-EBR) is one of its derivatives [100,101]. Enhancing the production of ROS is beneficial in cancer therapy by induction of the extrinsic and intrinsic pathways of apoptosis [102,103]. 13-EBR uses the same strategy for breast cancer therapy. Treating MDA-MB-231 breast cancer cells with 13-EBR is associated with the elevated concentration of ROS. As a consequence, an increase occurs in the level of pro-apoptotic factor BAX, while anti-apoptotic factor Bcl-2 undergoes down-regulation. Besides, 13-EBR enhances the levels of caspase-3, caspase-9 and poly (ADP-ribose) polymerase (PARP) showing the mitochondrial-induced apoptosis [104]. Lycorine is another plant-derived chemical belonging to the *Amaryllidaceae* plant family [105–107]. Administration of lycorine substantially reduces mitochondrial membrane potential (MMP) that is accompanied by the release of Cyt C into cytosol leading to apoptosis induction [96]. On the other hand, Rho-associated protein kinase (ROCK) contributes to the regulation of important biological processes such as cell proliferation, cell differentiation and cell apoptosis [108,109]. ROCK1 is involved in cancer progression and its inhibition occurs during cancer metastasis [110,111]. Lycorine stimulates mitochondrial-mediated apoptosis by up-regulation of ROCK1 [112]. Taking everything into account, it seems that naturally occurring compounds are able to target mitochondrial function in the induction of apoptosis in cancer cells [113,114].

Mitochondria play a significant role in energy production and regulation of cell death. Any impairment in the homeostasis of mitochondria can adversely affect the OMM resulting in releasing cyt C and induction of apoptotic cell death. Notably, the inhibition of mitochondrial dysfunction is of interest in the treatment of pathological conditions such as NDs and renal fibrosis to prevent further damage to these organs. However, the story is completely different in cancer cells. In cancer therapy, agents targeting mitochondria are applied to impair the homeostasis of this intracellular organelle leading to the stimulation of apoptotic cell death and decreased viability and proliferation of cancer cells.

## Regulation of mitochondria by plant-derived chemicals

Nowadays, the application of naturally occurring compounds has undergone an increase for management of various chronic diseases [115,116]. This is due to the great pharmacological impacts of plant-derived chemicals that have made them appropriate options in the treatment of a variety of disorders such as cancer, diabetes [117], cardiovascular diseases [118], autoimmune diseases [119] and infectious diseases [120]. There have been efforts to reveal the underlying mechanism of action of these agents [3,121]. It has been demonstrated that plant-derived chemicals are capable of affecting molecular signaling pathways and organelles in the treatment of diseases [3,4,115,121]. For instance, resveratrol can affect the ER to exert its therapeutic impacts [116]. This story is also similar for curcumin [62], berberine [80], emodin [122] and other natural products. So, it is conspicuous that organelles can be considered as potential targets of these natural products.

In the current section, we discuss the impact of naturally occurring compounds on mitochondria to direct the next sections about the special relationship of resveratrol and mitochondria in cancer therapy. Curcumin is one of the most important components of *Curcuma longa* with excellent therapeutic impacts including anti-tumor [123], hepatoprotective [124], cardioprotective [125], anti-diabetic [126], antioxidant [127] and anti-inflammatory [128]. Newly published articles have shed some light on the ability of curcumin in targeting mitochondria. Curcumin administration is considered as a promising strategy in cancer therapy by targeting mitochondria, impairing MMP, induction of the intrinsic pathway of apoptosis and consequently, reducing the viability and proliferation of cancer cells [129]. The plant-derived chemicals are well-known due to their great antioxidant impacts. It has been shown that high doses of natural antioxidants function as inducers of oxidative stress. In acute lymphoblastic leukemia, the administration of curcumin enhances the generation of ROS, leading to the activation of the intrinsic pathway of apoptosis via induction of mitochondrial dysfunction [130]. In addition to cancer therapy, the administration of curcumin is beneficial in the treatment of NDs by targeting mitochondria [131].

Emodin is an anthraquinone derivative with pharmacological effects such as antioxidant, anti-inflammatory, anti-tumor and neuroprotective [127]. Emodin supplementation is suggested to be beneficial in the treatment of colon cancer by regulation of molecular signaling pathways such as MAPK/JNK, PI3K/Akt, NF-κB and STAT to impair MMP, leading to the reduced viability and growth of tumor cells through mitochondrial-dependent manner [132]. Besides, accumulating evidence demonstrates that the side effects of emodin may be a result of emodin impact on mitochondria. The administration of emodin is associated with apoptotic cell death in hepatocytes by enhancing ROS levels and impairing MMP [133].

Berberine is another naturally occurring compound with great therapeutic and biological activities in clinical trials, *in vivo* and *in vitro* experiments [134,135]. The effects of berberine on mitochondria are associated with anti-tumor and protective activities of berberine. Co-delivery of berberine and paclitaxel is advantageous in cancer therapy, and promotes the efficacy of chemotherapy, since berberine selectively targets mitochondria, enhances the concentration of ROS, resulting in stimulation of apoptotic cell death [136]. Furthermore, berberine supplementation is a promising strategy in the amelioration of neurotoxicity from amyloid beta-mediated mitochondrial dysfunction [137] and oxidative stress-induced mitochondrial impairment [138]. These studies highlight the fact that mitochondrial dysfunction is beneficial in cancer therapy and using naturally occurring antioxidants such as curcumin, emodin, berberine and particularly, resveratrol can reduce the viability and growth of tumor cells by stimulation of mitochondrial dysfunction.

## Resveratrol affects mitochondria in cancer therapy

### Resveratrol and oxidative stress

Notably, plant-derived chemicals are usually applied for their high antioxidant activity. This is due to the potential role of ROS in the development of cancer [139,140]. However, the application of the high amount of these antioxidant agents is associated with their conversion to pro-oxidants resulting in the production of high levels of ROS [141,142]. This feature is used in cancer therapy. On the other hand, the enhanced concentration of ROS and consequently, the stimulation of oxidative stress displays tumor suppressor and oncogene activity [143]. A variety of molecular pathways contribute to the association of oxidative stress with cancer development or cancer suppression. It has been demonstrated that the interaction of ROS and non-coding RNAs is involved in cancer progression and inhibition. This dual role of oxidative stress and its function as a double-edged sword should be considered in treatment of cancer. Resveratrol uses same strategy in combating HepG2 cells. Administration of resveratrol enhances the concentration of ROS in cytosol of cancer cells. By upregulation of Bax and down-regulation of Bcl-2, the integrity of OMM is disrupted. Then, cyt C releases into the cytosol to form Apaf1-procaspase-9 complex resulting in apoptotic cell death [144]. In fact, resveratrol stimulates apoptotic cell death through improving the oxidative phosphorylation [145]. The studies approve that the high concentration of resveratrol or long-term exposure to resveratrol is related to a perturbation in the oxidative-antioxidative balance leading to apoptotic cell death [146–150].

### Resveratrol and apoptosis

Apoptosis is still a common way in reducing the viability and proliferation of cancer cells. However, there are concerns about this kind of cell death. Although apoptosis is beneficial in cancer therapy, there are controversial discussions showing that induction of apoptosis may be related to the enhanced proliferation of other surviving cancer cells. This phenomenon is known as apoptosis-induced proliferation (AIP) [151,152]. So, before making any decision in treatment of cancer, all the consequences should be considered to have an efficient chemotherapy. As it was mentioned, resveratrol uses various strategies to stimulate apoptosis in cancer therapy. One of them is making an increase in the level of oxidative stress that in turn, releases cyt C into cytosol resulting in apoptosis. However, resveratrol is capable of stimulation of mitochondrial dysfunction. Cisplatin is one of the chemotherapeutic agents that is extensively applied in the field of cancer therapy. Resveratrol is able to sensitize tumor cells to cisplatin therapy. Administration of resveratrol triggers mitochondrial dysfunction by mitochondrial membrane potential loss resulting in the induction of apoptosis in tumor cells [45]. Besides, resveratrol administration is associated with reduced viability and malignancy of prostate cancer cells by disruption of mitochondrial DNA [153]. Overall, cancer cells prefer to use glycolysis for the generation of ATP and simultaneously, enhance their glucose uptake known as Warburg effect [154]. Accumulating data demonstrates that the anti-tumor activity of resveratrol is a consequence of reducing the glucose metabolism of cancer cells [155–157]. In colon cancer cells, administration of resveratrol alters the metabolism into fatty acid oxidation to stimulate oxidative stress. This study highlights the potential role of mitochondrial respiration induction by resveratrol in cancer therapy [158]. However, the anti-tumor activity of resveratrol against various cancer cells is different. Resveratrol demonstrates highest anti-tumor activity againt leukemia cells mediated through mitochondrial dysfunction. This high anti-tumor activity is a result of mitochondrial-mediated apoptosis. Investigation of molecular pathways revealed that the administration of resveratrol down-regulates Akt resulting in decreased invasion and malignancy of leukemia cells [159]. These studies demonstrate that resveratrol is capable of the induction of apoptotic cell death in cancer cells via mitochondrial-mediated apoptosis [160–167].

Tumor necrosis factor-related apoptosis-inducing ligand (TRAIL) is one of the potential candidates in the stimulation of apoptotic cell death in tumor cells. The apoptosis is induced via attachment of death-inducing signaling complex into death receptors DR4 and DR5. Next, this complex binds to the Fas-associated protein with death domain and caspase-8 to activate caspase-9 and consequently, caspase-3 resulting in stimulation of apoptosis [168,169]. There is a close connection between TRAIL-mediated apoptosis and mitochondria. It seems that cyt C and Smac/Diablo released from mitochondria regulate TRAIL-mediated apoptosis [170]. One of the beneficial impacts of TRAIL is that this pathway induces apoptosis in tumor cells without harmfully affecting normal cells. However, some of the cancer cells acquire resistance into TRAIL-mediated apoptosis that in turn, significantly reduces the efficacy of agents activating the TRAIL pathway in cancer therapy. Hence, there have been efforts to sensitize cancer cells into TRAIL-mediated apoptosis. Resveratrol has demonstrated great potential in this case. Administration of resveratrol is accompanied by a loss of OMM and an elevation in ROS production that releases cyt C into the cytosol. This mitochondrial dysfunction is associated with a decrease in Bcl-2 and Bcl-xl in NSCLC cells leading to the sensitization of cancer cells into TRAIL-mediated apoptosis. Investigation of molecular signaling pathways demonstrated that resveratrol reduces the expression of the Akt/NF-κB signaling pathway to sensitize cancer cells into TRAIL-mediated apoptosis [171].

### Resveratrol and mitochondrial fusion and fission

Although much emphasis was put on either enhancing the ROS generation or induction of mitochondrial dysfunction, targeting other dynamic aspects of mitochondria is also beneficial in cancer therapy. Mitochondria play a significant role in cell growth and cell division. The fusion and fragmentation of mitochondria occur in G1/S and G2/M, respectively. Notably, cell growth can be negatively affected by the induction of fusion or inhibition of fission since mitochondrial fragmentation is vital for cell growth [172–175]. Mitofusin-2 (Mfn2) is one of the most important proteins that accelerates mitochondrial fusion [176]. Resveratrol up-regulates the expression of Mfn2 to stimulate mitochondrial fusion, leading to the decreased viability and proliferation of cancer cells [177]. Triggering mitochondrial fission is another promising strategy in cancer therapy. Similar to mitochondrial fusion, induction of fission is associated with an impairment in cell division and cell growth. Resveratrol administration significantly enhances the expression of microRNA (miR)-326 to down-regulate PKM2 resulting in mitochondrial fission that in turn, decreases the viability and proliferation of tumor cells [178].

### Resveratrol and Ca^2+^ signaling

There is a close connection between the ER and mitochondria. Due to the high loading of proteins into the ER for correct folding, this organelle needs a high amount of ATP produced by mitochondria [179,180]. As a consequence, the transportation of Ca^2+^ occurs from ER into mitochondria to promote the ATP generation [181]. Next, produced ATP can translocate into the ER to provide its energy demand [182]. It seems that the transportation of Ca^2+^ is a vital step in the ER and mitochondrial connection [183]. Accumulating data demonstrates that mitochondria are capable of regulating the Ca^2+^ concentration and high levels of Ca^2+^ negatively affect the potential of OMM [184–186]. A combination of resveratrol and piceatannol remarkably enhances the uptake of Ca^2+^ by mitochondria. Decreased expression of sarco/endoplasmic reticulum Ca^2+^ ATPase (SERCA) facilitates the Ca^2+^ uptake by mitochondria of cancer cells, leading to the stimulation of cell death [187].

### Resveratrol and sirtuin signaling pathway

Sirtuins are NAD^+^-dependent proteins that play a significant role in the regulation of biological processes in cancer cells such as metabolism and genome structure [188]. Sirtuin 1 (Sirt1) is one of the key members of the sirtuin family and its induction is associated with diminished viability and proliferation of tumor cells [189,190]. There is a bidirectional interaction between Sirt1 and AMP-activated protein kinase (AMPK) pathway. The forkhead box O3 (FoxO3) and LKB1 are involved in the SIRT1/AMPK axis. It has been reported that the SIRT1/AMPK axis is of importance in mitochondrial biogenesis, fission, fusion, and mitophagy [191]. Administration of resveratrol considerably reduces the viability and mitochondrial respiration of breast cancer cells, while it stimulates cell cycle arrest. It was found that resveratrol exerts these anti-tumor impacts by affecting mitochondrial respiration through Sirt1 up-regulation [192].

### Resveratrol and autophagy

Autophagy is a catabolic process that provides energy during starvation by the degradation of aged or damaged organelles and components [193]. Autophagy inhibition is widely observed in NDs such as AD [194]. However, autophagy plays a dual role in cancer cells. The increasing evidence demonstrates both the inhibitory and stimulatory impact of autophagy activation on the progression and viability of cancer cells [195,196]. Based on the most recent studies, autophagy stimulation is suggested to be advantageous in cancer therapy and enhancing the potential of chemotherapy [197]. However, other recent studies shed some light on the fact that autophagy is a protective mechanism and elevates the viability and proliferation of cancer cells [198,199]. Also, another study shows that the stimulation of autophagy is related to the reduced viability of tumor cells [200]. Furthermore, there is a close relationship between autophagy and apoptosis. The cells induce autophagy to ameliorate stress and look at autophagy as a protective and pro-survival mechanism. When the autophagy process is no longer able to maintain the survival of cells, the cells undergoing stress stimulate apoptosis through different pathways, for instance through ER stress. Resveratrol leads to the mitochondrial dysfunction by making damage to mtDNA that in turn, stimulates autophagy. Inhibition of autophagy enhances the mitochondrial-mediated apoptosis by resveratrol in colon cancer cells [201], showing the pro-survival role of autophagy. Signal transducers and activator of transcription 3 (STAT3) is a signaling pathway involved in the progression and malignancy of cancer cells [202]. Resveratrol has demonstrated great potential in the regulation of the STAT3 signaling pathway [203,204]. Resveratrol treatment enhances the level of cyt C in the cytosol to induce apoptotic and autophagic cell death. These anti-tumor effects are mediated by mitochondrial dysfunction through STAT3 inhibition [205].

### Resveratrol and NAF-1 protein

The nutrient-deprivation autophagy factor 1 (NAF-1) protein is a vital protein located on the OMM and ER [206]. NAF-1 plays a significant role in maintaining the integrity and function of mitochondria [207]. Besides, NAF-1 is associated with longevity since its expression undergoes down-regulation in elder mice than younger mice [208]. NAF-1 deficiency triggers mitochondrial impairment leading to autophagy induction for removal of damaged organelles [206]. Accumulating data demonstrates that NAF-1 functions as a prognostic signature and its up-regulation occurs in cancer cells [209,210]. Resveratrol targets NAF-1 protein in the treatment of pancreatic cancer cells. Resveratrol enhances ROS generation to activate the Nrf2 signaling pathway, leading to a diminution in the expression of NAF-1. The down-regulated NAF-1 stimulates apoptotic cell death, resulting in decreased viability and proliferation of pancreatic cancer cells [211].

### Resveratrol, glycolysis and mitochondrial oxidative phosphorylation

One of the hallmarks of cancer cells is their changed cellular metabolism. Cancer cells prefer to use glycolysis for ATP production and in this way, they elevate their glucose uptake to trigger glycolysis, a phenomenon known as Warbrug effect [154]. In a variety of cancer cells lines such as ovarian and breast cancers, resveratrol is able to stimulate apoptosis and diminish the viability of cancer cells via decreasing glucose metabolism [155–157]. So, in suppressing the proliferation and malignancy of cancer cells, resveratrol induces metabolic reporgramming. The administration of resveratrol shifts cancer cell metabolism from glycolysis into mitochondrial respiration. This is followed by mitochondrial recruitment, stimulation of mitochondrial respiration, fatty acid oxidation, enhancing ROS production and subsequenct activation of apoptosis via intrinsic pathway in cancer cells [158]. As it was mentioned, cancer cells apply Warbrug effect to produce daughter cells and enhance their growth via generation of ATP, NADPH and anabolic building blocks [212–214]. It was first believed that Warbrug effect occurs in cells that have mitochondrial oxidative phosphorylation defect, but it seems that Warbrug effect also occurs in cells having competent oxidative phosphorylation [215]. Attention has been directed toward suppressing glucose fermentation and triggering oxidative phosphorylation to inhibit cancer growth [216,217]. The hypoxia inducible factor-1 (HIF-1) is suggested to be involved in Warbrug effect and its inhibition is a promising strategy in cancer therapy [218,219]. In prostate cancer cells, resveratrol supplementation induces apoptosis and cell cycle arrest by shifting cellular metabolism from glycolysis into mitochondrial oxidative phosphorylation. Resveratrol improves mitochondrial biogenesis, fusion and respiration by HIF-1α down-regulation to stimulate cell cycle arrest in cancer cells [220]. These studies highlight the fact that resveratrol is able to target cellular metabolism of cancer cells and by enhancing oxidative phosphorylation predisposes cancer cells into apoptosis. Table 1 summarizes the effect of resveratrol administration on mitochondria in cancer therapy.

**Table 1:** Mitochondrial regulation by resveratrol in cancer therapy

Cancer cell line	Dose	Duration	Outcomes	Ref
B16F10 cells	0.1, 0.2, 0.5, 1, 2 and 10 μg/ml	24 h	Enhancing ROS generation subsequently induction of mitochondrial-mediated apoptosis	[221]
Mouse colon cancer cells CT26 tumor-bearing nude mice	2 mg/kg	5 h 35 days	Releasing cyt C into cytosol activation of caspase 3 and apoptotic cell death	[222]
Hepg2 cells xenografts in nude mice	1, 4, 8, 12 and 16 μg/ml	50 days	By down-regulation of PI3K/Akt pathway, Res-loaded NPs induced mitochondrial-mediated apoptosis	[223]
NCI-H460 cell line	-	24 h	Induction of mitochondrial-mediated apoptosis through NF-κB down-regulation	[224]
A549 cells	0.01, 0.1, 1, 5, 10, 20 and 50 μg/ml	24 h	Stimulation of apoptotic cell death	[225]
HepG2 (human hepatoma cells) and L-02 (human normal liver cells)	75, 100, 150 and 200 μM	3 h	Enhancing the level of ROS and induction of mitochondrial-mediated apoptosis	[144]
Human triple-negative breast cancer (MDA-MB-231), estrogen-receptor-positive breast cancer (MCF-7), cervix cancer (SiHa, HeLa), lung cancer (A549) and osteosarcoma (Saos-2) cells, and non-cancer human umbilical vein endothelial cells (HUVEC)	0.01, 0.1, 1, 10 and 200 μM	48 h	Promoting ROS generation and triggering apoptotic cell death	[145]
Human lung cancer cell lines, H838 and H520	40, 50, 60 and 70 μg/ml	24, 48 and 72 h	Enhancing the anti-tumor activity of cisplatin through mitochondrial dysfunction	[226]
TRAMP-C1, TRAMP-C2 and TRAMP-C3 cells	50 and 100 μM	16 h	Disruption of mitochondrial DNA induces apoptosis	[153]
SW620 colon cancer cell line	0, 5, 10, 20, 40, 80 and 160 μM	48 h	Shifting into fatty acid oxidation induces oxidative stress and apoptosis in cancer cells	[158]
A549 and HCC-15 cells	12.5, 25 and 50 μM	12 h	Sensitizing cancer cells into TRAIL-mediated apoptosis by induction of mitochondrial dysfunction	[171]
PC3 cancer cells	10 μM	48 h	By promoting mitochondrial fusion, Res suppresses cell division resulting in reduced proliferation of cancer cells	[177]
HeLa and Ea.hy926 cells	100 μM	36 h	Enhancing Ca^2+^ uptake by mitochondria results in apoptotic cell death	[187]
Two breast cancer cell lines, MCF-7 and MDA-MB-231	10, 25 and 50 μM	24 and 48 h	Induction of mitochondrial-mediated apoptosis through Sirt1 up-regulation	[192]
HCT116 colon cancer cells, PC3 and LNCaP prostate cancer cells, and MDA-MB231 breast cancer cells	60 and 120 μM	0, 24 and 36 h	Inhibition of autophagy enhances mitochondrial-mediated apoptosis	[201]
Human cervical [76], colon (DLD1), breast (MCF-7), and liver (HepG2) cancer cell lines	0, 25, 50, 100, 150 and 200 μM	24 h	Triggering mitochondrial fission is related to reduced viability and proliferation of cancer cells	[178]
Human OC CAOV-3 cells	120 μM	48 h	Stimulation of autophagic and apoptotic cell death by inhibition of STAT3 and via the mitochondrial pathway	[205]
Prostate cancer cells	1, 10, 25, 50 and 100 μM	1, 3 and 4 h	By p66Shc-Ser66 phosphorylation and ERK1/2 dephosphorylation, Res induces mitochondrial oxidative stress and apoptosis to suppress the viability and proliferation of cancer cells	[227]
Human T-cell leukemia cell line, CEM; human lung carcinoma cell line, A549; Human T-cell leukemia cell line, Molt4; human cervix adenocarcinoma cell line, HeLa	1, 5, 10 and 20 μM	48 h	A novel Res based tubulin inhibitor reduces the malignancy of cancer cells by making a loss in mitochondrial membrane potential and apoptosis induction	[228]
Rat C6 glioma cell line	1, 10, 30, 50 and 100 μM	48 h	Stimulation of cell cycle arrest and apoptosis by targeting mitochondria and subsequently caspase-3	[229]
MCF-7, MDA-MB-231 and MCF-10A cells	50 μM	24 h	Induction of ROS-mediated mitochondrial dysfunction and consequently cancer cell death	[230]
Caco2 cells	10 μM	0, 8, 24 and 30 h	Inhibition of cancer cell growth by triggering glucose oxidation via increasing AMPK or CamKKB expression	[231]
HepG2 (human liver carcinoma) cell line	12.5, 25, 50, 100, 200, 400 and 800 μg/ml	3 days	Stimulation of apoptosis by upregulation of caspase-3 and caspase-9 through mitochondrial pathway	[232]
Three human oral squamous cell carcinoma (OSCC) cell lines, CAL-27, SCC15, and SCC25	0, 50, 100, 200, 300, 400 and 500 μM	72 h	By triggering mitochondrial apoptosis, Res inhibits EMT	[233]
MCF7 and MDA-MB-231 cell lines	20 μM	72 h	Induction of apoptosis through the mitochondrial pathway	[234]
Cancer cell lines Panc-1, Mia paca-2, CF pac-1, and BxPC-3	0, 50, 100, 150 and 200 μM	24 h	By down-regulation of NAF-1, Res sensitizes cancer cells into apoptosis	[211]
HeLa human cervical cancer line	0, 5, 10, 20 and 40 μmol/l	48 h	Stimulation of apoptosis through the mitochondrial pathway	[235]
Human breast cancer cell lines MCF-7 and MDA-MB-231 and lung cancer cell line H1299	100 μM	24 h	Induction of apoptosis by reducing mitochondrial membrane potential	[236]
SCLC H446 cells	0, 10, 20, 30 and 40 μg/ml	24 h	Sensitizing cancer cells to cisplatin therapy through the mitochondrial pathway of apoptosis	[237]
Prostate cancer cells	0, 10, 20, 30, 40 and 50 μM	48 h	Res-loaded PLGA NPs enhanced ROS and induced mitochondrial-mediated apoptosis	[238]
Caco-2 cells (HTB-37TM)	0.1, 1, 10, 25, 50 and 100 μM	48 h	Targeting mitochondrial membrane potential and induction of intrinsic pathway of apoptosis	[237]
DBTRG glioblastoma cells	50 μM	24 h	Enhancing the efficacy of paclitaxel in chemotherapy by mitochondrial dysfunction and induction of apoptotic cell death	[239]
Glioblastoma A172 cell	0, 1, 5, 10 and 20 μm/ml	-	Exposing cells into resveratrol is associated with a decrease in expression of AMPK and YAP. Inhibition of PAK2 enhances ROS production and inhibits AMPK/YAP axis, resulting in mitochondrial-mediated apoptosis	[53]

## Selective targeting of mitochondria in cancer therapy using nanoparticles (NPs)

Nanotechnology is a multidisciplinary field that can be used in different fields such as engineering, medicine and chemistry [240–242]. Nanoparticles are structures with size less than 100 nm with capability of enhancing the bioavailability of drugs by encapsulating them and protection against degradation [8,243]. Also, they can reduce the clearance of drugs by escaping from phagocytosis system. Nanoparticles have been used in the field of cancer therapy due to their capability in selective targeting cancer cells and enhancing the accumulation of anti-tumor drugs in cancer cells. Nanoparticles can be used for delivery of both genes and drugs in cancer therapy [244]. There have been efforts to target mitochondria using NPs. Notably, due to the negative charge of mitochondrial membranes of tumor cells, these NPs should have positive charges [245,246]. It seems that mitochondrial-targeted NPs have great therapeutic impacts [247–249]. Liposomes are one of the most important nanocarriers that can be applied in the delivery of small molecules and genes [250,251]. Liposomes have a number of great properties such as high biocompatibility and capability of encapsulating both hydrophilic and hydrophobic drugs [252,253]. Surface modification of liposomes can make them specific for targeting mitochondria. Dequalinium (DQA) and 4-carboxybutyl triphenylphosphonium bromide (TPP) are cations that can be used for surface modification of liposomes resulting in the preparation of liposomes with the capability of targeting negatively charged mitochondria [254–257]. Mitochondriotropic liposomes are promising candidates in enhancing the delivery of resveratrol into mitochondria. Resveratrol-loaded nanocarriers effectively deliver resveratrol into mitochondria of cancer cells. Resveratrol promotes the generation of ROS to depolarize the mitochondrial membrane resulting in apoptotic cell death [221]. Loading resveratrol on the mitochondrial-targeted titanium disulfide nanosheets is another potential strategy in cancer chemotherapy. These nanosheets effectively target mitochondria leading to the enhanced delivery of resveratrol. After exposure to near-infrared irradiation, the releasing of resveratrol occurs. Resveratrol diminishes the mitochondrial membrane potential to release cyt C into the cytoplasm. Subsequently, caspase 3 was induced by caspase 9 resulting in the stimulation of apoptotic cell death [222].

Gold and silver NPs are extensively used in biomedicine due to their minimal toxicity and high biocompatibility [258,259]. Gold NPs have demonstrated great potential in both immunotherapy and cancer therapy [260,261]. Loading resveratrol on gold NPs improves its anti-tumor activity both *in vitro* and *in vivo*. Resveratrol-loaded gold NPs significantly accumulate in mitochondria and mediate apoptosis. It was found that resveratrol-loaded gold NPs elevate the expression of caspase-8 and BAX, while they reduce the expression of pro-caspase-9 and pro-caspase-3. Investigation of molecular signaling pathways reveal that this mitochondrial-mediated apoptosis is induced by down-regulation of PI3K/Akt pathway [223].

**Figure 1:**
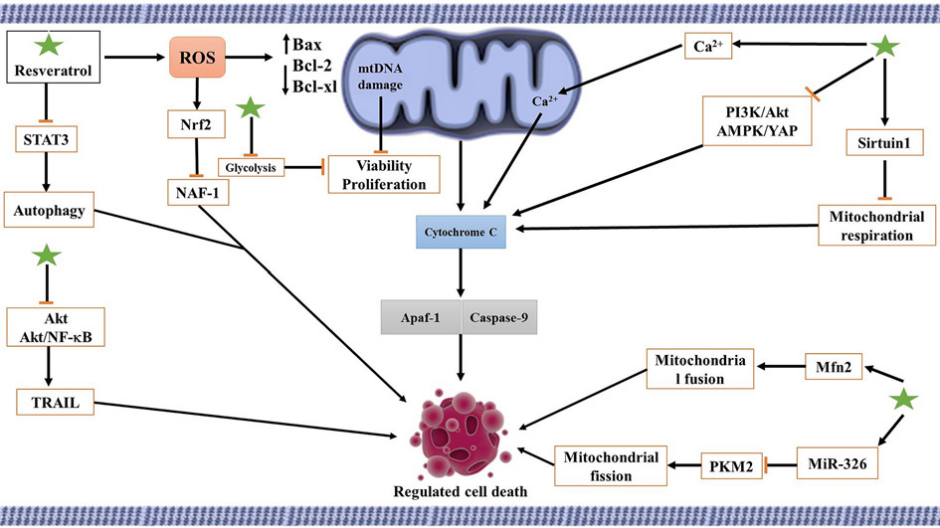
The involvement signaling network involved in stimulation of mitochondrial-mediated apoptosis by resveratrol

Polymeric NPs are capable of being used as nanocarriers for chemotherapeutic agents [262,263]. These NPs have shown great potential in accumulating in solid tumors via enhanced permeation retention (EPR) mechanism [264]. Resveratrol-loaded gelatin NPs can suppress the viability and proliferation of non-small cell lung cancer (NSCLC) cells by upregulation of BAX, p53, p21, caspase-3, and down-regulation of Bcl-2 and nuclear factor-κB (NF-κB) demonstrating the involvement of mitochondrial-mediated apoptosis [224]. NSCLC cells are sensitive to the mitochondrial-mediated apoptosis by resveratrol (Figure 1) [225]. Table 2 summarizes the nanocarriers used for delivery of resveratrol in targeting mitochondria in cancer therapy.

**Table 2:** Nanostrategies used for resveratrol delivery in targeting mitochondria in cancer therapy

Nanocarrier	Size (nm)	Zeta potential (mV)	Entrapment efficiency (%)	Cancer type	Major outcomes	Refs
Liposome	70	-9.78	82.58	Lung cancer	Selective targeting of mitochondria, enhancing the anti-tumor activity and induction of apoptotic cell death in cancer cells	[249]
Gelatin nanoparticle	294	-	-	Lung cancer	Stimulation of apoptosis and cell cycle arrest by down-regulation of Bcl-2 and NF-κB, and up-regulation of caspases due to targeted delivery	[224]
Gold nanoparticle	39	-32.5	-	Liver cancer	Delivering resveratrol in cells and mitochondria and subsequent activation of mitochondrial apoptosis	[223]
Titanium disulfide nanosheet	123	-37.2	-	Colon cancer	Higher cytotoxicity compared resveratrol alone by reducing mitochondrial membrane potential, releasing cytochrome *c* and induction of intrinsic pathway of apoptosis	[222]
Polymeric nanoparticle	231.12	86.18	50-70	Prostate cancer	Resveratrol-loaded nanoparticles significantly enhance the ROS production in cancer cells and stimulate mitochondrial-mediated cell death	[238]

## Conclusion and remarks

Plant-derived chemicals have demonstrated great potential in cancer therapy due to their capability in targeting various molecular pathways. In respect to the role of mitochondria in the induction of the intrinsic pathways of apoptosis, targeting this intracellular organelle is of importance in cancer therapy. Resveratrol is a naturally occurring compound that is capable of significantly reducing the viability and proliferation of cancer cells. Resveratrol is able to target mitochondria in cancer therapy. Primarily, resveratrol administration at a high amount enhances the concentration of ROS in mitochondria to stimulate mitochondrial dysfunction via making changes in the potential of OMM and opening the pores. As a consequence, cyt C releases into cytoplasm that triggers the caspase cascade resulting in apoptosis. A number of molecular signaling pathways are involved in mitochondrial regulation by resveratrol such as NF-κB and Sirt1. A challenge faced in cancer therapy is the resistance of cancer cells into chemotherapy and resveratrol treatment improves the potential of chemotherapeutic agents by sensitizing cancer cells to apoptotic cell death. These effects of resveratrol on mitochondria remarkably reduce the viability and proliferation of cancer cells and inhibit colony formation by tumor cells. One of the common features of cancer cells is their uncontrollable growth and proliferation. Resveratrol can target cell cycle and stimulates cell cycle arrest in G1/S and G2/M phases of cell cycle by regulation of mitochondrial fusion and fission. Resveratrol induces mitochondrial fusion and fission via Mfn2 upregulation and PKM2 down-regulation, respectively, leading to reduced viability and proliferation of cancer cells via cell cycle arrest. Resveratrol elevates Ca^2+^ uptake by mitochondria to impair its proper function and sensitize cancer cells into apoptosis. Resveratrol positively affects Sirt1 expression to diminish mitochondrial respiration and viability of cancer cells. Resveratrol is able to provide connection between apoptosis and autophagy in cancer cells. By inhibition of STAT3, resveratrol stimulates apoptosis and autophagy in cancer cells to reduce their progression. Suppressing autophagy enhances resveratrol-mediated apoptosis in cancer cells. As a protein located on OMM, NAF-1 inhibition by resveratrol sensitizes cancer cells into apoptosis. In order to enhance the potential of resveratrol in targeting mitochondria and induction of apoptosis, NPs can be applied and several studies have shown the higher anti-tumor activity of resveratrol compared to resveratrol alone due to targeted delivery. This review discussed the different aspects of resveratrol and mitochondrial in cancer therapy. All of the studies are in agreement with efficacy of resveratrol in activation of mitochondrial-mediated apoptosis by targeting various molecular pathways. However, there are controversial data about the effect of resveratrol on mitochondrial oxidative phosphorylation. Some of the studies demonstrate that resveratrol impairs mitochondrial oxidative phosphorylation, while others hold the view that resveratrol enhances mitochondrial oxidative phosphorylation. Both of them are in line with that fact that resveratrol targets oxidative phosphorylation to induce apoptosis. However, more studies are needed to clarify the discussed issues.

## Abbreviations

13-EBR: 13-ethylberberine
Aβ: amyloid-β
AD: Alzheimer’s disease
AMPK: AMP-activated protein kinase
Apaf-1: apoptotic protease activating factor 1
ATP: adenosine triphosphate
Bak: Bcl-2 antagonist/killer protein
Bax: Bcl-2 associated x protein
Bcl-2: B-cell lymphoma 2
cyt C: cytochrome *C*
DM: diabetes mellitus
DQA: dequalinium
EMT: epithelial-to-mesenchymal transition
EPR: enhanced permeation retention
ER: endoplasmic reticulum
FADD: Fas-associated protein with death domain
FoxO3: forkhead box O3
IMM: inner mitochondrial membrane
lncRNA: long non-coding RNA
Mfn2: mitofusion-2
miR: microRNA
MMP: mitochondrial membrane potential
MOMP: mitochondrial outer membrane permeabilization
mtDNA: mitochondrial DNA
NAF-1: nutrient-deprivation autophagy factor 1
ND: neurological disorder
NF-κB: nuclear factor-κB
NP: nanoparticle
Nrf2: nuclear factor erythroid 2-related factor 2
NSCLC: non-small cell lung cancer
OMM: outer mitochondrial membrane
PAK2: P21-activated protein kinase 2
PARP: poly (ADP-ribose) polymerase
PD-1: programmed death-1
PTX: paclitaxel
Res-SLNs: Res-loaded solid lipid nanoparticles
ROCK: Rho-associated protein kinase
ROS: reactive oxygen species
SERCA: ssarco/endoplasmic reticulum Ca^2+^ ATPase
Sirt1: sirtuin 1
STAT: signal transducer and activator of transcription
TPP: 4-carboxybutyl triphenylphosphonium bromide
TRAF6: TNF-receptor associated factor 6
TRAIL: tumor necrosis factor-related apoptosis-inducing ligand
YAP: Yes-associated protein
